# Reduced resting-state gamma-band power correlate with unaltered glutamate + glutamine levels in patients at clinical-high risk of psychosis

**DOI:** 10.1192/j.eurpsy.2024.1280

**Published:** 2024-08-27

**Authors:** A. S. Tomyshev, I. Lebedeva, A. Dudina, P. Menshchikov, D. Kupriyanov, M. Omelchenko

**Affiliations:** ^1^Laboratory of Neuroimaging and Multimodal Analysis, Mental Health Research Center; ^2^Clinical Science, LLC Philips Healthcare Russia; ^3^Department of youth psychiatry, Mental Health Research Center, Moscow, Russian Federation

## Abstract

**Introduction:**

There is growing evidence of excitation / inhibition (E/I) balance abnormalities in schizophrenia, which might be associated with abnormal gamma frequency oscillations and glutamate concentrations. However, to the best of our knowledge, only one multimodal study have examined such associations between EEG and metabolite characteristics in patients at clinical-high risk of psychosis (CHR) so far.

**Objectives:**

We aimed to investigate potential associations between GLX (glutamate + glutamine) levels and resting-state gamma-band power in CHR individuals and healthy controls (HC).

**Methods:**

Twenty right-handed male patients (16-27 years, mean age 19.9 ± 2.7) fulfilling CHR criteria and 19 healthy male controls (16-27 years, mean age 21.6 ± 3.6) underwent resting-state EEG (16 leads; 10−20 system) and MR spectroscopy at 3T MRI scanner with voxels of 30×30×30mm located in left and right medial prefrontal cortex. Spectral analysis with estimation of gamma-band power (30-45 Hz) were conducted. MEGA-PRESS acquisitions were analyzed with jMRUi (ver. 5.1 Alpha), levels of GLX were calculated as a ratios to creatine + phosphocreatine (GLX/Cr). Gamma-band (30-45 Hz) spectral power and GLX/Cr were compared between groups. Correlations between EEG and metabolite data were analyzed with regression model including age and chlorpromazine equivalents as covariates.

**Results:**

Compared to healthy controls, patients showed reduced spectral gamma-band power in 6 leads (Table). No alterations in GLX/Cr were detected. Positive correlations between altered gamma-power in all leads (except Cz) and GLX/Cr in left medial prefrontal cortex were revealed in CHR (F3: r=0.51, p=0.006; F8: r=0. 54, p=0.004; C3: r=0.37, p=0.037; Pz: r=0.51, p=0.039; P4: r=0.56, p=0.009). No correlations in HC group were found. Chlorpromazine equivalents did not correlate with GLX/Cr of gamma power in CHR group.

**Table.** Results of between-group comparisons corrected for multiple comparisons

Lead	CHR Mean±SD	HC Mean±SD	p -value	F	Cohen’s d	Cohen’s d CI 95%
F3	0.97±0.62	1.4±0.64	0.0097	7.2	-0.69	-1.22 -0.16
F8	0.84±0.61	1.45±1.03	0.0072	7.8	-0.71	-1.24 -0.19
C3	0.97±0.55	1.44±0.64	0.0026	9.9	-0.79	-1.32 -0.27
Cz	1.03±0.61	1.42±0.52	0.0074	7.7	-0.70	-1.22 -0.18
Pz	1.17±0.7	1.62±0.63	0.0098	7.1	-0.68	-1.2 -0.16
P4	1.04±0.66	1.53±0.66	0.0051	8.5	-0.74	-1.27 -0.22

**Conclusions:**

The findings suggest that clinical-high risk of psychosis is associated with widespread alterations in resting-state gamma-band power. Positive correlations of such alterations with GLX/Cr and absence of such correlations in HC group are presumably indicative of disturbances in the excitation / inhibition balance in CHR individuals.

*This study was supported by RFBR grant 19-29-10040*

**Disclosure of Interest:**

None Declared

